# Modulation of paraoxonases during infectious diseases and its potential impact on atherosclerosis

**DOI:** 10.1186/1476-511X-11-92

**Published:** 2012-07-23

**Authors:** Ayman Samir Farid, Yoichiro Horii

**Affiliations:** 1 Department of Clinical Pathology, Faculty of Veterinary Medicine, Benha University, Moshtohor, Toukh, 13736, Qalyubia, Egypt; 2 Laboratory of Parasitic Diseases, Faculty of Agriculture, University of Miyazaki, Gakuen-Kibanadai, Nishi 1–1, Miyazaki 889-2192, Japan

**Keywords:** Atherosclerosis, Paraoxonases, Oxidative stress, Infectious diseases

## Abstract

The paraoxonase (PON) gene family includes three members, PON1, PON2 and PON3, aligned in tandem on chromosome 7 in humans and on chromosome 6 in mice. All PON proteins share considerable structural homology and have the capacity to protect cells from oxidative stress; therefore, they have been implicated in the pathogenesis of several inflammatory diseases, particularly atherosclerosis. The major goal of this review is to highlight the modulation of each of the PONs by infective (bacterial, viral and parasitic) agents, which may shed a light on the interaction between infectious diseases and PONs activities in order to effectively reduce the risk of developing atherosclerosis.

## Introduction

The desire to understand the correlation between infection, inflammation and oxidative stress in various diseases, including atherosclerosis, has captured the imagination of many investigators. The involved mechanisms are strictly regulated and possibly inter-connected in order to maintain oxidative homeostasis in cells and tissues. Understanding the biology and function of such mechanisms will pave the way for discovery of novel therapeutic agents in the fight against various inflammatory diseases.

The paraoxonases (PONs) comprise a family of closely related enzymes that includes PON1, PON2 and PON3, and these are aligned next to one another on chromosome 7 in humans and on chromosome 6 in mice. PONs share around 70% nucleic acid identities and are believed to be derived from a common precursor [1,2].

In this review, we focused on the effects of infectious agents on PONs, emphasizing its potential roles against infections and elucidating the relationship between infection and atherosclerosis.

### Physiological roles of PONs

PONs have different cell and tissue distributions, as well as different regulatory mechanisms, thus suggesting distinct physiological roles for each of them. These roles, however, remain largely unknown [3] especially in the light of the crucial fact that the physiological substrates of PONs remain still poorly known. Indeed, the most striking characteristic of PONs is their multitasking capacity, which allows PONs to play a role in several different pathways, not only limited to lipid oxidation metabolism but also including the intriguing field of innate immunity.

### PON1

PON1 is the most studied member of the PON family and much of our understanding of the PON enzymes is derived primarily from studies involving PON1 protein. In humans, the PON1 gene is mainly expressed in the liver, giving a protein product of 354 amino acids with a molecular mass of 43–45 kDa, and is released into normal circulation [4]. There is growing evidence from experimental, clinical and epidemiological studies that underscores the role of PON1 in protection against atherosclerosis [5]; however, the precise mechanisms remain elusive.

The enzymatic activity for which the enzyme is named is screened by using synthetic substrates without regard for the native substrate or its role in human (patho)biology [6]. The ability of PON1 to hydrolyze paraoxon was employed as a method to measure PON1 activity in several species and tissues. PON1 paraoxonase enzymatic activity can be modulated by polymorphisms in the PON1 gene locus, including the Q192R polymorphism, in which glutamine (Q) is replaced by arginine (R) at position 192. The Q isoform has low activity in hydrolyzing paraoxon, while the R isoform shows high activity [4]. PON1 also possesses arylesterase activity, with phenyl acetate being one of its best substrates. Furthermore, PON1 exhibits good lactonase activity; hydrolyzing a wide range of lactones [7].

Although PON1’s natural substrates are uncertain, thioester homocysteine (Hcy)-thiolactone, which is a product of an error editing reaction in protein biosynthesis formed when Hcy is mistakenly selected by methionyl-tRNA synthetase, is hydrolyzed to Hcy by PON1 [3,4]. Therefore, it has been suggested that PON1 should be properly named homocysteine-thiolactonase [8]. Hcy is a risk factor for the development of cardiovascular disease [9]. Proposed mechanisms underlying Hcy pathobiology include protein modification by Hcy-thiolactone, oxidative stress, inflammation, autoimmune response, endothelial dysfunction, and thrombosis [10]. Thus, the Hcy-thiolactonase activity of PON1 is likely to contribute to the cardioprotective role of PON1.

**Figure 1:**
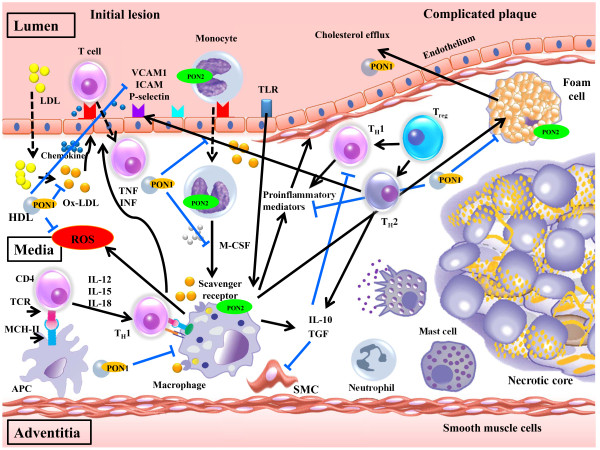
**A proposed mechanism for atherosclerosis development and the roles of PON1 and PON2 against atherosclerosis.** Moving from left to right, low-density lipoprotein (LDL) diffuses from the blood into the innermost layer of the artery. The LDL in the artery wall is modified by oxygen radicals to oxidized LDL (oxLDL) which in turn induces endothelial cells to express leukocyte adhesion molecules, such as vascular cell-adhesion molecule-1 (VCAM1), intercellular adhesion molecule-1 (ICAM-1), and P-selectins. Monocytes and T cells bind to adhesion molecules-expressing endothelial cells and respond to locally produced chemokines by migrating into the arterial tissue. Monocytes differentiate into macrophages in response to local macrophage colony-stimulating factor (M-CSF) and other stimuli. Scavenger receptors mediate macrophage uptake of ox-LDL particles, which leads to intracellular cholesterol accumulation and the formation of foam cells. Ox-LDL and other ligands promote the production of many pro-inflammatory molecules by macrophages. T cells undergo activation after interacting with antigen-presenting cells (APCs), such as macrophages or dendritic cells. A T helper 1 (T_H_1)-cell-dominated response ensues, possibly owing to the local production of interleukin-12 (IL-12), IL-18 and other cytokines. T_H_1 cells produce inflammatory cytokines including interferon-α (IFN-α) and tumour-necrosis factor (TNF). These cytokines and others prompt macrophage activation, production of other pro-inflammatory mediators, activate endothelial cells, increase adhesion-molecule expression and the propensity for thrombus formation, and stimulate proliferation and migration of smooth-muscle-cell as well as collagen production. Plaque inflammation might be attenuated in response to the anti-inflammatory cytokines IL-10 and transforming growth factor-α (TGF-α), which are produced by several cell types including regulatory T and T_H_2cells, macrophages, and for TGF-α, also vascular cells and platelets. The atherosclerotic lesion has a core of lipids, including cholesterol crystals, living and apoptotic cells and a fibrous cap with smooth muscle cells and collagen. Several types of cells of the immune response are present throughout the atheroma including macrophages, T cells, mast cells and DCs. HDL-associated PON1 inhibits the influx of cholesterol by oxidized LDL into macrophages by reducing Ox-LDL levels, reducing Ox-LDL uptake via the macrophage scavenger receptor, reducing macrophage-mediated oxidation of LDL, and increasing the hydrolysis of macrophage oxidized lipids. HDL-associated PON1 also inhibits macrophage cholesterol biosynthesis and enhances HDL-mediated cholesterol efflux. Monocyte/macrophage-associated PON2 also protects LDL against oxidation and reduces the oxidative stress caused by preformed ox-LDL. TCR; T-cell receptor, TLR; Toll-like receptor, MCH; Major histocompatibility complex, ROS; Reactive oxygen species.

Most serum PON1 is associated with the cholesterol-carrying high-density lipoprotein (HDL) (“good cholesterol”) through its retained N-terminal hydrophobic region. HDL is important for PON1 secretion and stabilization by HDL-associated apolipoprotein A-I; however, less than 5% of serum PON1 is also associated with chylomicrons and VLDL, but not LDL [11]. Several lines of evidence have suggested that PON1 protects against atherosclerosis by its evident ability to guard low-density lipoproteins (LDL) against oxidative stress, reduce macrophage foam cell formation, and prevent atherosclerosis development (Figure 1). In the artery wall, PON1 inhibits LDL oxidation, in doing so prevents the ox-LDL-induced up-regulation of monocyte chemoattractant protein-1 (MCP-1) production by endothelial cells [12,13]. MCP-1 displays chemotactic activity for monocytes into the intima that differentiate into macrophages. The latter take up ox-LDL in an unregulated manner to become foam cells [14], which leads to atherosclerotic plaque development [15,16]. Since the control of cholesterol efflux is of vital relevance for foam cell formation, PON1 also enhances cholesterol efflux from macrophages [17] and inhibits macrophage cholesterol biosynthesis [18]. Such roles suggest that PON1 has crucial effects on the initial steps of atherosclerosis.

The special localization of PON1 in the HDL complex of human serum led to speculation that the enzyme plays also an important physiological role in lipid metabolism and that it protects against the development of atherosclerosis [19]. This notion was confirmed by the capacity of PON1 to hydrolyze lipid peroxides, which prevents foam cell formation [20]. More interestingly, a recent study by Deakin et al. [21] showed that PON1 is not a fixed component of HDL can exert its protective function outside the lipoprotein environment since it can be transferred from HDLs to the external face of the plasma membrane of cells in an enzymatically active form conferring protection against oxidative stress.

Another important clue regarding the physiological function of PON1 has been provided by studies in mice lacking this enzyme. Shih et al. [22] found that PON1 “knockout” (KO) mice develop atherosclerosis when fed an atherogenic diet, and their HDL, in contrast to wild-type HDL, failed to prevent LDL oxidation in cultured artery wall cells. This study clearly established the anti-oxidative and anti-inflammatory potential of PON1 in vivo and also showed its potential role in the prevention of atherosclerosis. Moreover, mice with combined PON1/apoE KO exhibited more atherosclerosis than apoE KO mice and their LDL particles were more susceptible to oxidation [23]. Furthermore, PON1-deficient mice showed increased oxidative stress in macrophages, which could be related to the activation of cellular nicotinamide adenine dinucleotide phosphate (NADPH) oxidase and to a decrease in cellular reduced glutathione (GSH) content. On the other hand, purified PON1 directly reduced macrophage oxidative stress [18].

Owing to the development of genetically modified PON1 KO and transgenic (Tg) mice, the potential roles of PON1 in the context of macrophage functions could be studied. HDL from human PON1 Tg mice increased cholesterol efflux from mouse peritoneal macrophages (MPM) and the J774 A.1 macrophage cell line via the ATP-binding cassette (ABCA1) transporter, and the binding activity to the same macrophage cell types also increased. In addition, the presence of high PON1 levels in HDL induced the formation of lysophosphatidylcholine (LPC) in the macrophages, which is thought to increase HDL binding to macrophages and thus contributes to a more important cholesterol efflux via an apoA-I-mediated mechanism. These results indicate that PON1 status on HDL particles will influence binding to macrophages and cholesterol efflux, further demonstrating the beneficial effects of PON1 in the early stages of atherosclerosis [17,24].

Recently, the absence of PON1 in mice was associated with a broad array of vascular changes, including enhanced oxidative stress and thrombogenicity, as well as significant increases in leukocyte adhesion and mRNA levels of the aortic adhesion molecule P selectin and inter-cellular adhesion molecule-1 (ICAM1). Aortic superoxide production was also significantly higher in PON1 KO animals when compared with wild-type controls [25].

PON1 may confer protection against macrophage apoptosis under basal conditions via LPC formation and further by up-regulation of the macrophage scavenger receptor class B, type I (SR-BI)-mediated HDL binding to the cells. As macrophage apoptosis is an important feature of atherosclerotic plaque development, PON1 deficiency may lead to the enhanced atherosclerosis development observed in mice, as a result of reduced SR-BI-mediated HDL protection against apoptosis [26].

More recently, HDL-associated PON1 inhibits monocyte-to-macrophage differentiation. Monocyte-derived macrophages play a central role in the development of arterial foam cells and atherosclerotic lesions. Such action could lead to attenuation of macrophage foam cell formation and atherosclerosis development [27]. Therefore, PON1 is closely linked to the control of oxidative stress and inflammation, mainly at the circulation level, where its association with HDL particles is related to the prevention of atherosclerosis [25,28].

### PON2

Human PON2 is ubiquitously expressed and is found in various tissues with primary localization in the plasma membrane, which suggests that its functions are distinct from those reported for PON1 and PON3 [29]. While little is known about the physiological or pathophysiological role of this protein, PON2 has been reported to possess anti-oxidant properties. In addition to its ability to reduce the oxidative stress caused by preformed mildly oxidized LDL, decreasing LDL-mediated induction of inflammatory response in cells [29], PON2 can also protect LDL against oxidation [30]. Thus, one function of PON2 may be to act as a cellular anti-oxidant, protecting cells from oxidative stress.

Animal studies have shown that mice subjected to adenovirus-mediated expression of PON2 (AdPON2) have an increased anti-oxidant capacity with lower levels of lipid hydroperoxides when compared to mice treated with either PBS or empty vector. Although PON2 is not normally found in circulation and was not detected in the serum of these mice, its effect may be induced by modulating the properties of circulating lipoproteins, for example, affecting the susceptibility of LDL to oxidation and the capacity of HDL to protect LDL against oxidation [31].

On the other hand, when subjected to a high-fat diet for 15 weeks, PON2-deficient animals developed significantly larger (2.7-fold) atherosclerotic lesions when compared with controls. Moreover, LDL isolated from these animals was more susceptible to oxidation and induced a greater degree of monocyte chemotaxis. Furthermore, there was enhanced macrophage trafficking into the artery wall in PON2-deficient mice, as determined by macrophage staining in aortic sections using CD68 as a marker. When macrophages were isolated from PON2-deficient mice, they exhibited both higher levels of oxidative stress and enhanced pro-inflammatory properties, as well as showing increased tumor necrosis factor (TNF)-*α* and interleukin (IL)-1*β* gene expression after LPS-induced inflammation [32].

PON2 may exert significant protection against macrophage triglyceride (TG) accumulation, macrophage TG biosynthesis, microsomal diacylglycerol acyltransferase 1 (DGAT1) activity and macrophage oxidative stress, in the presence and absence of glucose [33,34] (Figure 1). PON2 gene and protein expression have been detected in various parts of the human gastrointestinal tract [35], and the addition of purified PON2 to permeabilized intestinal Caco-2 cells protects against iron-ascorbate-induced oxidative stress [36]. Surprisingly, PON2 protein was detected on the apical (luminal) side of Caco-2 culture medium, raising the possibility that the intestinal cells are capable of secreting PON2 into the intestinal lumen, where it may perform another, as yet unclear function [35], possibly against infectious agents.

### PON3

PON3 was the last of the paraoxonases to be characterized. Draganov et al. [37] were the first to purify and characterize rabbit plasma PON3. Several studies then demonstrated that PON3 protects against oxidation and inflammation, thus suggesting that PON3 is atheroprotective [5,38,39]. Draganov and his colleagues reported that rabbit PON3 purified from serum was capable of inhibiting copper-induced LDL oxidation in vitro to a greater degree than rabbit PON1 [37]. Reddy et al. [40] showed that pretreatment with cultured human aortic endothelial cells with supernatants from HeLa Tet On cell lines overexpressing PON3 prevents the formation of mildly oxidized LDL and inactivates preformed mildly oxidized LDL. Rosenblat et al. [30] demonstrated the presence of PON3 in murine macrophages, but not human macrophages, which suggests that mouse PON3 influences atherogenesis more directly through its expression in artery wall cells.

AdPON3 in 26-week-old apolipoprotein E-deficient mice was also shown to protect against atherosclerosis, with mice showing significantly lower levels of serum lipid hydroperoxides and enhanced potential for cholesterol efflux from cholesterol-loaded macrophages. In addition, LDL was less susceptible to oxidation, whereas HDL was more capable of protecting against LDL oxidation. These results confirmed that although human PON3 in mice did not reside in HDL particles, the reduction in atheroma is mediated by the ability of PON3 to enhance the anti-atherogenic properties of plasma [41].

A study by Shih et al. [42] demonstrated that overexpression of human PON3 decreases atherosclerotic lesion formation in transgenic mice (C57Bl6/J and LDLRKO background; 55% and 34% reduction, respectively), in a male-specific fashion. In addition, male PON3 Tg mice maintained on either low-fat chow or high-fat Western diet exhibited decreased adiposity when compared with age and diet-matched, male non-Tg littermates. Moreover, this study showed that elevated human PON3 expression decreased obesity in male mice. These findings suggest a protective role for PON3 against atherosclerosis and obesity.

One of the interesting physiological functions of all three PONs is the ability, via lactonase activity, to hydrolyze and inactivate bacterial quorum sensing (QS). QS molecules are extracellular signals secreted by Gram-negative bacteria to regulate biofilm formation and secretion of virulence factors [43,44]. Of the three PONs, PON2 appears to have the highest activity against the QS factors. The second member to have evolved is proposed to be PON3, followed by PON1 [7]. This function of PONs indicates their potential importance as novel components of innate immunity.

These findings clearly demonstrate the important protective roles played by PONs against inflammation and oxidative stress. It is possible that PON2 fulfills these crucial functions in various organs, whereas HDL-associated PON1 and PON3 primarily act in blood circulation.

### Mutual relationship between PONs and infections

Numerous risk factors are involved in the development of atherosclerosis, such as hypertension, cigarette smoking, diabetes, hyperlipidemia and hypercoagulability [45]. However, as many as 50% of patients with atherosclerosis lack the abovementioned risk factors, which suggests that there are additional factors predisposing individuals to atherosclerosis [46,47].

There are multiple epidemiological studies to support the notion that infections can be considered risk factors for atherosclerosis. The paradigm that infection by bacteria and/or viruses is a risk factor for atherosclerosis via direct infection of vascular cells or via the indirect effects of cytokines or acute-phase proteins induced by infection at non-vascular sites [48] emphasizes the “infectious hypothesis” of atherosclerosis. This relates to current atherogenesis theories that accept the crucial role of inflammation in the development of atherosclerotic plaques [49]; however, the role of some kind of infections (like parasitic infection) on atherosclerosis and related anti-atherogenic mechanisms (including PONs) remains uncertain.

### PONs and bacterial infections

Experimental studies have indicated that PON1 activity is altered during the acute-phase response. LPS injection, which mimics Gram-negative infections, in mice increases serum amyloid A (SAA) through nuclear factor-*κ*B (NF-*κ*B) transactivation and decreases apoA-I and PON1 by inhibiting peroxisome proliferator-activated receptor (PPAR)-*α* activation. TNF-*α*, IL-1*β* and IL-6 mediate these changes through stimulation of hepatocytes [50]. Bin Ali et al. [51] also found that LPS induces a further 50% decrease in hepatic PON1 mRNA in male mice and moderate increases in female mice through pro-inflammatory cytokine (IL-1*β* and TNF-*α*)-unmediated pathways. However, these pro-inflammatory cytokines have been shown to up-regulate (IL-6) or down-regulate (IL-1*β* and TNF-*α*) PON1 gene expression in HepG2 human hepatoma cells [52].

Similarly to previous results, mice with 18 G cecal ligation and puncture (CLP) to induce slow leakage of intestinal flora in the abdominal cavity exhibit gradual onset sepsis that closely mimics human sepsis. Plasma paraoxonase activity decreases up to 24 hours post-CLP in association with increased IL-6 and decreased HDL levels, and PON1 activity is positively correlated with total anti-oxidant activity. The cause-effect relationship between decreased PON1 activity and increased oxidative stress has not been established, but it is most likely a dynamic bi-directional relationship [53].

Human clinical studies have shown that in septic patients there are significant decreases in plasma PON1 (paraoxonase and arylesterase) activity, and this is negatively correlated with C-reactive protein (CRP), which is produced in response to the oxidizing environment induced by sepsis. This increased binding of free radicals to PON1 accounts for the decrease in PON1 activity in the circulation [54].

More recently, Naderi et al. [55] showed that patients with pulmonary tuberculosis have significantly lower paraoxonase and arylesterase activities when compared with healthy subjects. This reduction is most likely due to imbalance of oxidant/anti-oxidant systems in pulmonary tuberculosis patients, as supported by the findings of Nezami et al. [56], who found decreased levels of total anti-oxidant capacity, red blood cell superoxide dismutase activity and whole blood glutathione peroxidase activity with increased levels of malondialdehyde in pulmonary tuberculosis cases, thus suggesting a higher susceptibility of LDL to oxidation and higher levels of lipid peroxidation. This environment clearly provides a higher risk for atherosclerosis.

At the same time, epidemiological studies in humans indicated that infection by *Helicobacter pylori*, a potential cause of atherosclerosis, significantly decreases serum paraoxonase and arylesterase activities. This decrease may be attributed to decreases in HDL-C and, in part, to increased oxidative stress and inflammatory conditions induced by *H. pylori* infection [57].

*Chlamydia pneumoniae* is an obligate intracellular bacterium that causes acute and chronic respiratory disease in humans and is associated with an increased risk of cardiovascular disease [58,59]. Infection of mice with *C. pneumoniae* reduces serum PON1 activity and the anti-inflammatory properties of HDL by repressing gene expression via serum amyloid A elevation [50]. In addition, acute infection is associated with an increase in the frequency of intra-plaque hemorrhage [60]. These results indicate that *C. pneumoniae* contributes to the progression and destabilization of atherosclerotic lesions.

Interestingly, the expression of human PON1 in transgenic *Drosophila* results in increased resistance to infection by *Pseudomonas aeruginosa* via inactivation of the QS factor *N*-(3-oxododecanoyl)-L-homoserine lactone (3OC12-HSL) of *Pseudomonas*[61]. In vitro studies using PON1 KO mouse serum have shown that PON1 is important for degradation of 3OC12-HSL [61] through its lactonase activity [7,62]; thus, playing an important role in the fight against bacterial biofilm formation [63]. Importantly, chronic *P. aeruginosa* infection in the lung can stimulate atherogenesis in the aorta and coronary artery under a cholesterol-supplemented diet [64]. As PON1 has the ability to inactivate QS in Gram-negative bacteria, it is possible that under physiological conditions, PON1 (mostly with PON2 and PON3) can prevent the bacterial colonization associated with several pro-inflammatory factors, including QS molecules and atherogenic lipids [65]. These results indicate that PON1, in addition to its anti-atherogenic role, can also be considered part of the innate immune system [66].

While little is known about its role, PON2 appears to have the highest activity against QS factors. As the pulmonary system is a primary site of infection for *P. aeruginosa*, experiments using airway epithelial cells cultured from PON2-KO mice and a QS reporter strain of *P. aeruginosa* confirmed a two-fold increase in QS. This indicates that deficiency of PON2 impairs 3OC12-HSL degradation by airway epithelial cells and that diffusion of 3OC12-HSL into airway cells is the rate-limiting step for degradation of the molecule, irrespective of bacterial density [67]. Thus, PON2 expression does not appear to affect the growth of *P. aeruginosa*, but degrades the bacterial QS signal.

Knockdown of PON2 by transfecting cells with small-interfering RNA (siRNA) in human aortic endothelial cells treated with 3OC12-HSL or oxidized 1-palmitoyl-2-arachidonoyl-sn-glycero-3-phosphocholine (Ox-PAPC) resulted in increased pro-inflammatory response (IL-8, COX2, IL-1*β* and ICAM-1), apoptosis markers and unfolded protein response (UPR) [68]. This indicates that anti-atherogenic effects of PON2 include destruction of QS molecules.

The *P. aeruginosa* QS signal 3OC12, which is inactivated by PON2 [67], has the ability to down-regulate PON2 mRNA, protein and hydrolytic activity in A549 and EA.hy 926 cell cultures. These decreases were at least partly mediated by increases in cytosolic Ca^2+^, which mediates the degradation of PON2 protein and mRNA [69]. The hydrolytic activity of PON2 was decreased much more extensively and rapidly than the protein, indicating a likely post-translational event that blocks the hydrolytic activity of PON2. These findings not only support a role for PON2 in the defense against *P. aeruginosa* virulence, but also reveal a potential mechanism by which the bacterium may subvert the protection afforded by PON2 [70].

The third member of the PON family, PON3, is expressed in the skin, salivary gland, glandular epithelium of the stomach, intestine, liver hepatocytes, pancreatic acinar cells, heart, adipose tissue and bronchiolar epithelium, with differences in distribution patterns between humans and mice [35,39,71]. To date, there has been relatively little learned about PON3. However, in addition to its anti-atherogenic and anti-obesity effects [41,42], PON3 is known to hydrolyze bacterial QS molecules, such as 3OC12-HSL [7,63]; therefore, it is plausible that the presence of PON3 plays a protective role against bacterial infection.

### PONs and viral infections

Several epidemiological studies have assessed the association between viral infection and the development of atherosclerosis (Table 1). In the late 1970s, experimental infection of germ-free chickens with an avian herpesvirus induced an arterial disease that resembled human atherosclerosis [72].

**Table 1:** Potential infectious causes of atherosclerosis and/or underlying diseases

**Infection**	**Host**	**Affected PONs**	**Effect on PONs**	**Mediator(s)**	**Atherosclerotic effect/heart attack**	**References**	
**Bacterial infections**							
LPS	Human	PON1	↓	Oxidative stress and oxidative modification of HDL	**+**	[53,54]	
	Mice (male)	PON1	↓	· Decrease hepatic PON1 synthesis · Oxidative stress	**+**	· [51,73] · [53]	
	(female)	PON1	↑↓	· Increased hepatic PON1 synthesis (in case of ↑) · Oxidative stress (in case of ↓)	**+**	· [51] · [53,74]	
	Syrian hamster	PON1	↓	(TNF-*α* and IL-1)-mediated down-regulated hepatic PON1 mRNA		[75]	
	HepG2 human hepatoma cells	PON1	↓	(IL-1*β* and TNF-*α*)-mediated hepatic PON1 down-regulation		[52]	
	HepG2 human hepatoma cells	PON1	↑	(IL-6)-mediated hepatic PON1 up-regulation		[52]	
	Pulmonary tuberculosis	Human	PON1	↓	Oxidant/anti-oxidant systems imbalance	**+**	[55]
	*Helicobacter pylori*	Human	PON1	↓	Oxidative stress and oxidative modification of HDL	**+**	[57]
	*Chlamydia pneumoniae*	Mice	PON1	↓	Down-regulate hepatic PON1 mRNA	**+**	[50]
	*Pseudomonas aeruginosa*	A549 and EA.hy 926 cell cultures	PON2	↓	Down-regulate PON2 mRNA		[69]
**Viral infections**							
Influenza A strain WSN/33	C57BL/6 J mice	PON1	↓	Decreased hepatic PON1 protein synthesis	**+**	[76]	
	Hepatitis C virus (HCV)	Human	PON1	↓	Oxidant/anti-oxidant systems imbalance	**+**	[77]
	HCV	Human	PON3	↑	Oxidant/anti-oxidant systems imbalance	**+**	[78]
	Hepatitis B virus (HBV)	Human	PON1	↓	· Oxidant/anti-oxidant systems imbalance · Decrease (dysfunction) of HDL · Decrease (dysfunction) of ApoA-1		· [79] · [80] · [81,82]
	Human immunodeficiency virus (HIV)	Human	PON1	↓	Decrease (dysfunction) of HDL	**+**	[83,84]
	HIV	CD34^+^CD4^+^ hematopoietic cell line TF-1 and thymocytes derived from the human fetal conjoint thymus/liver hematopoietic organ of SCID-hu mice	PON2	↑	Up-regulation of cellular PON2 mRNA expression		[85]
HIV	Human	PON3	↑	Oxidative stress	**+**	[86]	
**Parasitic infections**							
*Trypanosoma cruzi* (Chagas’ disease)	Human	PON1 PON2 PON3	±	· Oxidative stress, apoA-I miss structure · T_H_1 immune polarization	±	· [87] · [88-91]	
*Nippostrongylus brasiliensis*	Rat	PON1	↓	Pro-inflammatory cytokines-mediated hepatic PON1 down-regulation	±	[92-94]	
*Trichinella spiralis*	Rat	PON1	↓	Oxidant/anti-oxidant systems imbalance	±	[95]	
*Schistosoma mansoni*	Mice	Hepatic PON1	↓	Oxidative stress	**—**	[96]	

The arrows ↑ and ↓ represent increased and decreased PONs activity.

The symbols +, — and ± mean, respectively, increase, decrease and unknown effect on atherosclerosis.

Experimental study has shown that intranasal inoculation of influenza A strain WSN/33 in C57BL/6 J mice results in significant decreases in the activities of paraoxonase and the platelet-activating factor acetylhydrolase, which reached their lowest levels by day 7 after infection. This was associated with lower HDL anti-inflammatory properties and increased monocyte/macrophage trafficking into arteries. If this is the case in human infection, these changes might explain the increased risk for heart attack and stroke after influenza infection [76].

Hepatitis C virus (HCV) is a major cause of viral hepatitis. There are approximately 170 million people worldwide who are chronically infected by this virus. Infection by HCV does not typically resolve, and nearly 80% of infected individuals become chronic carriers who may then progress to severe liver diseases [97]. HCV infection is associated with increased oxidative stress, which is marked by an increase in oxidants and a decrease in anti-oxidant capacity of the cells [97]. In addition to the contribution by chronic inflammation caused by infection, direct induction of reactive oxygen species/reactive nitrogen species (ROS/RNS) and mitochondrial dysfunction by the virus is likely.

In cell culture systems, HCV expression, replication and infection can induce oxidative stress [98-100]. Subsequently, oxidative stress has been identified as a significant mechanistic pathway culminating in the development of hepatic damage [101]. As PON1 exerts a protective effect against oxidative stress, it is plausible that there is an association between this enzyme and liver impairment. A study by Ali et al. [77] confirmed that there were significant decreases in PON1 (paraoxonase and arylesterase) activity in chronic and cirrhotic HCV patients with higher serum nitric oxide levels and myeloperoxidase activity. These results are consistent with those of Ferré et al. [102], who studied rats with carbon tetrachloride-induced fibrosis and showed decreased PON1 activity and an inverse correlation with lipid peroxidation, while the addition of zinc as an anti-oxidant was associated with enhanced PON1 activity and normalization of lipid peroxidation. These results suggest that PON1 activity is involved in the defense against free radical production in liver organelles.

Although PON1 enzyme activity is a more important factor in atherosclerosis and coronary heart disease than PON1 genotype [103,104], it is interesting to observe that there was a higher frequency of the RR isoform of the 192 polymorphism in healthy subjects than in those with chronic HCV infection [105]. This is supported by the results of Aviram et al. [106], who found that the PON1Q allele appears to be more efficient than the PON1R allele in hydrolyzing lipid peroxides in both coronary and carotid lesion homogenates. Mackness et al. [103] also showed that the R allele is associated with a modest increase in the risk of coronary heart disease.

On the other hand, serum PON3 concentration, in patients with chronic hepatic impairment as a consequence of HCV infection, is significantly elevated when compared with control subjects, and its concentration is related to the severity of the periportal alterations and to serological markers of anti-apoptosis, thus suggesting an anti-apoptotic role for PON3 [78].

Another type of hepatitis caused by viral infection, hepatitis B, also showed lower serum paraoxonase and arylesterase activities in chronic active hepatitis B patients when compared with inactive carriers and control individuals [80]. This observation is supported by the results of Schulpis et al. [79], who found decreased paraoxonase and arylesterase activities in mothers with HBV disease, mostly due to the liver damage and low total anti-oxidant capacity. This reduction of serum PON1 activity during HBV infection may be the result of changes in synthesis or secretion of HDL [107], and significant decreases [81] and post-transcriptional modification of nascent ApoA-1 [82]. It is likely that PON1 protects HDL from oxidation and this is likely to be related to the attributed HDL-anti-apoptotic function [79]; therefore, PON1 may contribute to the protective effects of HDL in maintaining lower levels of HBV DNA [108].

Several prospective and retrospective studies have established the association between human immunodeficiency virus (HIV) infection and atherosclerotic coronary artery disease [109]. Inflammation has been recognized as the key pathologic process leading to early atherosclerosis. Patients with HIV have an enhanced state of inflammation. Several specific pathways of inflammation linking HIV infection to increased cardiovascular risk have been elucidated. HIV-infected individuals have higher CRP values and higher circulating concentrations of the adhesion molecules intercellular adhesion molecule-1 (ICAM-1) and vascular cell adhesion molecule-1 (VCAM-1), as compared with uninfected individuals [110,111].

There are several key changes in lipoprotein metabolism in the course of HIV infection, including increased lipid peroxidation, hypocholesterolemia and hypertriglyceridemia, and decreased HDL concentration [83]. This explains the significant decrease in serum PON1 activity in HIV-infected patients [84]. Anti-retroviral therapy using non-nucleoside reverse transcriptase inhibitors such as nevirapine increases HDL concentration and apoA-I production. Concomitantly, modest increases in lecithin:cholesterol acyltransferase and cholesteryl ester transfer protein activity are also observed [112]. It is likely that apoA-I increases the stability and activity of PON1 [113] in treated patients, which may contribute to the beneficial effects of high HDL concentration in HIV-infected patients.

Changes in PON1 activity play a role in the course of HIV infection, which is an area that is worthy of further investigation. PON1 may also play an anti-infective role, as this enzyme increases cholesterol efflux from the cell, as well as the binding of the HDL particle to its receptor (ABCA1) [17]. Membrane metabolism is modulated by the efflux of cholesterol to the HDL particle, and this phenomenon would influence HIV replication, as the virus requires cholesterol rafts in the plasma membrane for final assembly and entry into the cell. In addition, there is a positive association between serum PON1 activity and CD4+ T lymphocyte count and its serum concentration with *β*-2-microglobulin; the latter being an effective marker of HIV infection activity [84].

In contrast to PON1 activity during HIV infection, both in vitro and in vivo studies have shown increased PON2 activity and up-regulation of cellular PON2 mRNA expression upon HIV-1 infection in the CD34^+^CD4^+^ hematopoietic cell line TF-1 and in thymocytes derived from the human fetal conjoint thymus/liver hematopoietic organ of SCID-hu mice. HIV-1 infection results in dephosphorylation of STAT5 in the absence of granulocyte-macrophage colony stimulating factor (GM-CSF), and this is associated with increases in PON2 gene expression, activity and protein levels, thus indicating that PON2 is part of the innate immune response to viral infections [85].

On the other hand, PON3 concentrations also increase significantly (about three times) in HIV-infected patients with respect to controls and are inversely correlated with oxidized LDL levels, which indicates that PON3 plays a protective role against oxidative stress and increased lipid peroxidation in HIV infection [86]. Long-term use of non-nucleoside reverse transcriptase inhibitor (NNRTI)-based anti-retroviral therapy is associated with a decrease of PON3 concentrations. NNRTI promotes anti-atherogenic changes in HDL form and function, including normalization of size and lipid composition and enhancement of reverse cholesterol transport, and induces higher PON1 activity [114,115]. These effects, together with the increased concentration before treatment, indicate that PON3 is not associated with the presence of sub-clinical atherosclerosis in HIV-infected patients, although lipid peroxidation and atherosclerosis are known to be strongly linked to such infection [116]. Therefore, PON3 is in some way involved in protection against HIV infection [86].

### PONs and parasitic infections

In the battle against parasitic infection, host immune response is central, but this also carries a cost. For example, generation of oxidative stress is an important factor in immune activation [117]. The generation of oxidants during parasitic infection occurs via three routes: first, they are released by immune cells that use their cytotoxic effects to kill the pathogen; second, oxidants are by-products of oxygen consumption, and increased metabolic activity during an immune response may contribute to the generation of additional toxic oxidants; and third, parasites themselves can be directly responsible for oxidant release through degradation products of their own metabolism. While useful in immune protection, non-targeting toxic oxidants have a potentially important negative side-effect by damaging host tissues and obstructing their function [118].

Considering the “oxidative modification hypothesis” of atherogenesis [16,119], together with the chronicity of several types of parasitic infection that can influence the host for years to decades [120,121], discussing the effects of parasitic infections and their associated immune responses on PON activity in the context of atherosclerosis remains of clinical importance.

The protozoan parasite *Trypanosoma cruzi* causes Chagas’ disease, which is a major endemic problem from the southern United States to temperate South America [122]. Infection invokes alteration in the microvascular and macrovascular circulation and severe cardiomyopathy [123]. Although epidemiological studies in humans did not show a direct linkage between Chagas’ disease and atherosclerosis [124], experimental studies, using in vivo and in vitro models, demonstrated cellular infiltration (CD8^+^ and CD4^+^) and associated cytokine (IL-4, IL-5, IL-6 and TNF-*α*) production in the heart [88,89]. Further studies showed increased expression of ICAM-1, IL-6 and TNF-*α* in aortic endothelial cells with associated inflammation in the adventitia consisting mainly of CD4^+^ and CD8^+^ T cells and macrophages [125]. A recent study indicated that immune response polarization to a T_H_1 response during experimental *T. cruzi* infection is responsible for the development of the chronic cardiac form of the disease [90,91].

On the other hand, *T. cruzi* infection was found to induce oxidative stress in the host indicated by increased levels of TBARS and SOD [87]. Interestingly, HDL meets some of the nutritional needs of *T. cruzi*, as supported by the observation that epimastigote growth is slowed significantly in vitro by lipid depletion and that transition of *T. cruzi* trypomastigotes to amastigotes is accompanied by a shift from carbohydrate to lipid-dependent energy metabolism [126,127]. Furthermore, exposure of cruzipain to human HDL during in vitro and in vivo infection in mice with *T. cruzi* generates several truncated apoA-I fragments [126]. Importantly, apoA-I, which is the major structural and functional protein component of HDL and is necessary for stabilizing and maintaining the optimum PON1 activity [113,128], was mostly truncated in *T. cruzi* in the sera of human patients when compared with controls [126]. Raper et al. [129] demonstrated the presence of apoA-I and PON1 in both trypanosome lytic factors (TLF1 and TLF2) in human serum. In humans, these factors confer resistance to infection with cattle *T. brucei brucei*. The authors suggested that the presence of apoA-I, regardless of its concentration, is important in the assembly of lytic particles, as supported by the finding that serum from an individual with familial apoA-I deficiency is not trypanolytic [130]. Further studies confirmed that PON1 influences survival in mice infected with *T. congolense*; mice overexpressing PON1 had significantly longer lifespans than wild-type mice, and mice deficient in PON1 had significantly shorter lifespans [131].

The possible mechanisms by which PON1 may influence trypanosome virulence may be related to the role of PON1 in the immune response. PON1 has been shown to have anti-inflammatory properties [13,132]. This is supported by the observed trypanosome survival of a relatively short period of time (less than 10 days) after infection, thus indicating that it may be function of innate, rather than adaptive, immunity [131]. PON1 also has multiple enzymatic activities, including esterase and lactonase, which may be important against trypanosome infection.

The effects of the second most common parasitic infections caused by nematodes on PON1 activity were studies by Farid et al. [92,93] who showed that infection by *Nippostrongylus brasiliensis,* a gastrointestinal nematode that infects mice and rats and has a similar life cycle as human pathogens *Ancylostoma duodenale* and *Necator americanus*[133], reduces serum PON1 activity in male rats. Studies by the same group showed that *N. brasiliensis* infection in rats fed a high-fat diet led to reduced serum PON1 activity in association with an atherogenic lipid profile [94].

At least two possible mechanisms can be postulated for the observed decrease in PON1 activity during *N. brasiliensis* infection. The infected rats had down-regulated hepatic PON1 expression, which is closely correlated with serum PON1 activity [4,75,134]. The mechanism by which hepatic PON1 mRNA is down-regulated during *N. brasiliensis* infection in rats is induction by various pro-inflammatory cytokines associated with that infection. This notion is supported by the up-regulation of hepatic IL-1*β*, IL-1*β* receptor (R), TNF-*α* and TNFR1 mRNA expression. These results are consistent with the observation of increased serum levels of pro-inflammatory cytokines (IL-1, IL-6 and TNF-*α*) on day 9 after infection with *N. brasiliensis*[92], which provides evidence that hepatic PON1 mRNA is down-regulated during *N. brasiliensis* infection in response to inflammatory conditions either in hepatic tissue or induced during larval migration. The role of pro-inflammatory cytokines in down-regulation of PON1 mRNA is primarily mediated by NF-*κ*B [50,52].

An alternative or complementary explanation is the increased levels of oxidative stress parameters (TBARS) [94] as a result of host immune-dependent damage to helminth parasites via a nonspecific defense reaction by the host [135]. These results in enhanced free radical production and oxidatively damaged macromolecules, and PON1 enzyme can be inactivated by these compounds [136]. This is supported by the results recently obtained by Mido et al. [95].

Another interesting finding was reported by Chelur et al. [137], who confirmed expression of PONs in the nematode *Caenorhabditis elegans* system. The expressed PONs are thought to interact with lipids, and this interaction may be crucial to the localization of the degenerin channel complexes to the specialized membrane domains mediating mechanotransduction in touch cells of the nematode.

The opposite appears to be the case for the third parasitic category of trematodes. The results obtained by Doenhoff et al. [138] showed that atherogenesis is reduced by approximately 50% when compared with uninfected controls in apoE^−^/^−^ mice subjected to low-intensity, chronic experimental infection with *Schistosoma mansoni*. The authors attributed this to parasite-mediated effects on lipid metabolism. As schistosomes do not synthesize cholesterol [139] and the parasite breaks down LDL [140] via inducible LDL receptors, this would account for the decrease in blood cholesterol levels in infected animals. Alternatively, schistosome infection may reduce circulating lipid levels by inhibiting lecithin:cholesterol acyltransferase (LCAT) activity [141]. The mechanism by which schistosomiasis reduces atherosclerotic lesion development appears to be independent of the development of a T_H_2 environment, as exposure to eggs induces a classic T_H_2 response (IL-4, IL-5 and IL-13 production) but does not alter lesion progression [142,143].

The involvement of T_H_2 cells in atherosclerosis is ambiguous, but the notion is supported by the work of Stanley et al. [144], who found that the lipid-lowering effects of parasite eggs may be induced as a consequence of a granulomatous reaction against tissue-embedded eggs, rather than a direct response to the eggs themselves. These results are supported by a study by Helmy et al. [96], who showed that serum and liver arylesterase and paraoxonase activities were significantly lower in mice at 10 weeks after infection with *S. mansoni*, as compared to uninfected healthy mice. However, these activities are partially restored in infected animals receiving zinc as an anti-oxidant, indicating that the changes in PON1 are influenced by oxidative stress associated with infection.

As there are no more detailed studies on the relationship between parasitic infection and PONs, particularly PON2 and PON3, further mechanistic investigations would be valuable in exploring the measures to counteract the inflammatory and oxidative processes in parasitic infections and in providing new insights into the roles played by these infections during atherogenesis regarding that the typical immune response in the initial steps of atherogenesis is T_H_1 [145-147]. Switching the immune response from T_H_1 to T_H_2, which occurs in some parasitic infections, may induce secretion of the anti-inflammatory cytokines, leading to slower progression of atherosclerotic lesion development [146,148].

## Conclusion

There is now growing evidence that PONs acting alone or in concert with other mechanistic pathways prevent/retard atherosclerosis development in vivo. Under conditions such as infection, the anti-inflammatory and anti-atherogenic properties of PONs are reduced by pro-inflammatory proteins and/or associated oxidative stress. Therefore, detailed biochemical, cell-based, animal and epidemiological studies are necessary to further identify the physiological roles of PONs and the molecular mechanisms by which PONs render their protective effects against atherosclerosis. Future studies on the potential implications of PONs activity modulation (e.g. by means of recombinant human PON engineered for specific molecular target) during infectious disease are of great importance. In addition, it is of particular interest to speculate on a broader host defense role for PONs against bacterial, viral and parasitic infections.

## Abbreviations

PON(1,2,3), paraoxonase-1,2,3; HDL, high-density lipoprotein; VLDL, very low-density lipoprotein; LDL, low-density lipoprotein; KO, “knockout”; NADPH, nicotinamide adenine dinucleotide phosphate; GSH, glutathione; Tg, transgenic; MPM, mouse peritoneal macrophages; ABCA1, ATP-binding cassette; LPC, lysophosphatidylcholine; ICAM1, inter-cellular adhesion molecule-1; SR-BI, scavenger receptor class B, type I; TNF, tumor necrosis factor; IL-, interleukin -; LPS, lipopolysaccharide; DGAT1, diacylglycerol acyltransferase 1; QS, quorum sensing; NF-κB, nuclear factor-κB; PPAR, peroxisome proliferator-activated receptor; 3OC12-HSL, N-(3-oxododecanoyl)-L-homoserine lactone; Ox-PAPC, oxidized 1-palmitoyl-2-arachidonoyl-sn-glycero-3-phosphocholine; COX2, cyclooxygenase-2; UPR, unfolded protein response; ROS/RNS, reactive oxygen species/reactive nitrogen species; TBARS, thiobarbituric acid reactive substance; SOD, superoxide dismutase.

## Competing interests

The authors declare that they have no competing interests.

## Authors’ contributions

ASF and YH conceived and designed the manuscript. ASF wrote the manuscript, YH reviewed the manuscript. All authors approved the final version of the manuscript.
